# Point‐of‐care assessment of C‐reactive protein and white blood cell count to identify bacterial aetiologies in malaria‐negative paediatric fevers in Tanzania

**DOI:** 10.1111/tmi.12823

**Published:** 2016-12-28

**Authors:** Helena Hildenwall, Florida Muro, Jaqueline Jansson, George Mtove, Hugh Reyburn, Ben Amos

**Affiliations:** ^1^ Global Health, Health Systems and PolicyDepartment of Public Health SciencesKarolinska InstitutetStockholmSweden; ^2^ Astrid Lindgren Children's HospitalKarolinska University HospitalStockholmSweden; ^3^ Kilimanjaro Christian Medical University CollegeMoshiTanzania; ^4^ Joint Malaria ProgrammeSt Augustine's HospitalMuhezaTanzania; ^5^ National Institute for Medical ResearchAmani CentreMuhezaTangaTanzania; ^6^ London School of Hygiene and Tropical MedicineLondonUK

**Keywords:** fever, children, infection, C‐reactive protein, leucocytes, point‐of‐care test

## Abstract

**Objective:**

To assess the role of point‐of‐care (PoC) assessment of C‐reactive protein (CRP) and white blood cell (WBC) count to identify bacterial illness in Tanzanian children with non‐severe non‐malarial fever.

**Methods:**

From the outpatient department of a district hospital in Tanzania, 428 patients between 3 months and 5 years of age who presented with fever and a negative malaria test were enrolled. All had a physical examination and bacterial cultures from blood and urine. Haemoglobin, CRP and WBC were measured by PoC devices.

**Results:**

Positive blood cultures were detected in 6/428 (1.4%) children and urine cultures were positive in 24/401 (6.0%). Mean WBC was similar in children with or without bacterial illness (14.0 × 10^9^, 95% CI 12.0–16.0 × 109 *vs*. 12.0 × 10^9^, 95% CI 11.4–12.7 × 109), while mean CRP was higher in children with bacterial illness (41.0 mg/l, 95% CI 28.3–53.6 *vs*. 23.8 mg/l, 95% CI 17.8–27.8). In ROC analysis, the optimum cut‐off value for CRP to identify bacterial illness was 19 mg/l but with an area under the curve of only 0.62. Negative predictive values exceeded 80%, while positive predictive values were under 40%.

**Conclusion:**

WBC and CRP levels had limited value in identifying children with bacterial infections. The positive predictive values for both tests were too low to be used as single tools for treatment decisions.

## Abstract (fr)

**Objectif:**

Evaluer le rôle de l’évaluation sur le point des soins de la protéine C‐réactive (CRP) et du taux de globules blancs pour identifier la maladie bactérienne chez les enfants tanzaniens souffrant de fièvre non paludique et non sévère.

**Méthodes:**

Dans le département ambulatoire d'un hôpital de district en Tanzanie, 428 patients âgés de 3 mois à 5 ans présentant de la fièvre et un test de paludisme négatif ont été inscrits. Tous ont eu un examen physique et des cultures bactériennes de sang et d'urine. L'hémoglobine, la CRP et le taux de globules blancs ont été mesurés par des dispositifs utilisés sur le point des soins.

**Résultats:**

Des cultures sanguines positives ont été détectées chez 6/428 (1,4%) des enfants et les cultures d'urine ont été positives chez 24/401 (6,0%). La moyenne des taux de globules blancs était semblable chez les enfants avec ou sans maladie bactérienne (14,0x10^9^ ; IC95%: 12,0 ‐ 16,0 contre 12,0x10^9^ ; IC95%: 11,4 ‐ 12,7) alors que la CRP moyenne était plus élevée chez les enfants atteints de maladie bactérienne (41,0 mg/L, IC95%: 28,3 ‐ 53,6 contre 23,8 mg/L, IC95%: 17,8 ‐ 27,8). Dans l'analyse ROC, la valeur seuil optimale pour la CRP pour identifier la maladie bactérienne était de 19 mg/l, mais avec une aire sous la courbe (AUC) de seulement 0,62. Les valeurs prédictives négatives dépassaient 80% alors que les valeurs prédictives positives étaient inférieures à 40%.

**Conclusion:**

Les taux de globules blancs et de CRP avaient une valeur limitée dans l'identification des enfants atteints d'infections bactériennes. Les valeurs prédictives positives pour les deux tests étaient trop faibles pour être utilisées comme des outils uniques dans les décisions de traitement.

## Abstract (es)

**Objetivo:**

Evaluar el papel de la evaluación en el punto de atención de la proteína C‐reactiva (PCR) y células blancas sanguíneas (CBS) para identificar enfermedades bacterianas en niños Tanzanos con fiebre no severa y no malárica.

**Métodos:**

En las consultas externas de un hospital distrital de Tanzania**,** se reclutaron 428 pacientes con edades entre los 3 meses y 5 años, que se presentaron con fiebre y una prueba negativa para malaria. A todos se les realizó una evaluación física y cultivos bacterianos de sangre y orina. Se midió la hemoglobina, PCR y CBS mediante pruebas diagnósticas rápidas en el punto de atención.

**Resultados:**

Se detectaron hemocultivos positivos en 6/428 (1.4%) niños y cultivos positivos en 24/401 (6.0%). La media del CBS era similar en niños con y sin enfermedad bacteriana (14,0x10^9^, IC 95% 12.0‐16.0 vs 12.0x10^9^, IC 95% 11.4‐12.7) mientras que la CBS media era mayor en niños con enfermedad bacteriana (41.0 mg/L, IC 95% 28.3‐53.6 vs 23.8mg/L, IC 95% 17.8‐27.8). En el análisis de la curva ROC, el punto de corte óptimo de la PCR para identificar la enfermedad bacteriana eran 19 mg/l pero con un área bajo la curva de solo 0.62. Los valores negativos predictivos excedían el 80%, mientras que los valores predictivos positivos estaban por debajo del 40%.

**Conclusión:**

Los niveles de CBS y PCR tenían un valor limitado a la hora de identificar a los niños con infecciones bacterianas. Los valores predictivos positivos para ambas pruebas eran demasiado bajas para utilizarse como herramientas únicas en la toma de decisiones.

## Introduction

Childhood febrile illnesses in resource‐poor settings are often treated presumptively with antibiotics and/or antimalarials due to the lack of diagnostic tools to differentiate between aetiologies. Since 2010, WHO recommend parasitological testing to guide antimalarial drug use wherever possible and the subsequent availability of malaria rapid diagnostic tests (RDTs) provides an opportunity for improved management of paediatric febrile illness 1, 2. However, inappropriate treatment of malaria‐negative patients continues 3. Where increased testing for malaria has been introduced, antibiotic prescribing is poorly targeted and often increased 4, 5, 6, in spite of evidence that many of these infections are viral 7, 8, 9.

Levels of C‐reactive protein (CRP) and white blood cells (WBC) can be assessed by point‐of‐care (PoC) tests that may differentiate between bacterial and viral illnesses, but results remain inconclusive 10, 11, 12, 13, 14. Despite these disparities in results, WBC and CRP are commonly used in high‐income settings and may be valuable to reduce numbers of blood cultures in hospitalised children or limit antibiotic use in outpatients 15, 16. To our knowledge, the potential for CRP and WBC to identify bacterial infections in non‐severe paediatric fevers has not been clarified in sub‐Saharan Africa.

## Methods

### Study area

Data collection took place at St Augustine's Hospital (formally Teule Hospital), Muheza District, Tanzania, from July 2011 until November 2012. St Augustine's Hospital primarily covers a rural population where malaria transmission is perennial with increased transmission during the two rainy seasons. There has been a considerable decline in the prevalence of *Plasmodium falciparum* in recent years 17. Patient residency in one of the 85 villages from which there had been more than 10 paediatric admissions to St Augustine's Hospital in 2006 was used to define the study area.

### Procedures

Patients were recruited at the hospital's Reproductive and Child Health clinic. Patients between 3 months and 5 years of age were consecutively recruited to enter a first and second screening. The first screening assessed children for presence of fever on that day or the day before, residency in the study area and absence of life‐threatening illness, as defined in the Integrated Management of Childhood Illness (IMCI) algorithm 18.

In the second screening, patients were excluded if they suffered from an obvious soft tissue infection, if they presented with a chronic condition except HIV infection – which was only recorded if reported by the patient's guardian – and if the patient had already been enrolled in the same study within the previous month. A negative rapid test for *P. falciparum* (Paracheck™; Orchid Biomedical, Mumbai) was required to enter the study.

If a child was eligible for enrolment, the study information was read to the patient's guardian, and after informed consent had been obtained, a clinical history and examination were recorded by a study clinical officer using the IMCI algorithm 18. Enrolled patients were asked to return to the hospital for scheduled follow‐up visits after 1 and 2 weeks to collect data on symptom progression as reported in a related publication 9.

### Laboratory procedures

A maximum of 10 ml of blood was drawn from all enrolled patients through sterile venepuncture using a butterfly and syringe. At least 3–6 ml of blood was aseptically inoculated into a BactALERT (BioMerieux, France) paediatric FAN aerobic culture bottle. All cultures with growth of a suspected or verified pathogen were immediately reported back to the clinicians to allow for treatment adjustments of affected patients. Detailed information on blood culture procedures and results is reported in a related publication 9.

Remaining venous blood was used for PoC measurement of haemoglobin (HemoCue™, Ängelholm, Sweden), CRP (Afinion AS100 Analyzer™; Axis‐Shield, Oslo, Norway) and white blood cell count (HemoCue™, Ängelholm, Sweden). CRP results less than 5 mg/l were reported by the machine as <5 mg/l, while results higher than 200 mg/l were reported as >200 mg/l. CRP results above 100 mg/l were repeated to confirm results but did not have any impact on management decisions.

Urine samples were obtained by clean catch collection at enrolment. Careful instructions for cleaning before sample collection were given to all caretakers to avoid skin pathogen contamination of urine samples. Urine was collected in sterile bottles and a Multistix dipstick was performed immediately upon receiving the urine. If nitrate‐ or leucocyte‐positive, the urine was sent for bacterial culture within 1 h of collection. A growth of 10^5^ colony‐forming units (cfu) per ml was considered significant bacteriuria. If a urine culture resulted in two or more colony types at greater than 10^5^/ml, it was reported as contaminated and a repeat specimen requested. *Bacillus* species and coagulase‐negative organisms other than *Staphylococcus saprophyticus* were reported as contaminants and not further identified.

### Data management and analysis

For the analysis of relations between CRP/WBC levels and bacterial infections, we defined potential bacterial infections as either a positive urine or blood culture or symptoms suggestive of IMCI pneumonia as defined in the IMCI algorithm during the study period, for example cough or difficult breathing with a respiratory rate of >40 breaths/min for children older than 1 year and >50 breaths/min for children below 1 year of age 18.

Data were manually double‐entered in MS Access and analysed using Dell Statistica version 13 and Stata 12 software. Proportions with confidence intervals were calculated for prevalence of clinical characteristics. Wilcoxon rank‐sum test was used to compare continuous variables between groups, and Mann–Whitney *U*‐test with receiver operator characteristic was used to identify cut‐off values for CRP/WBC to predict bacterial illness.

### Ethics

Written informed consent to participate was obtained from the parent or legal guardian of each child in the study. The Ethical Review Boards of the National Institute for Medical Research in Tanzania approved the study including the consent procedures.

## Results

**Figure 1:**
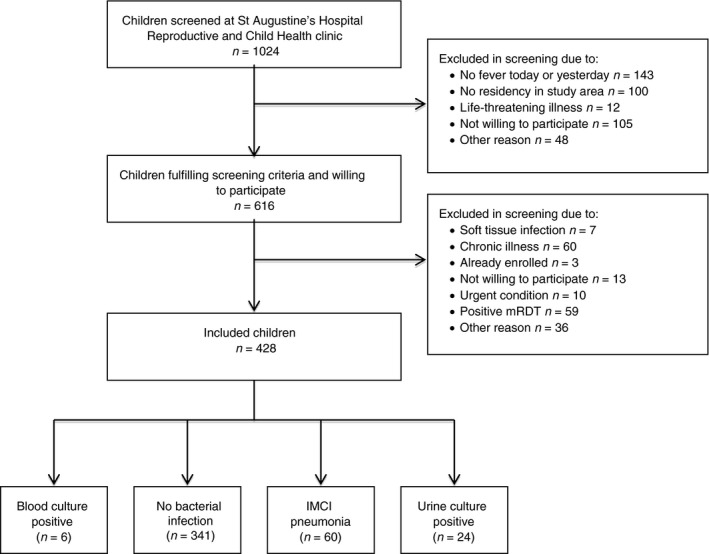
Study flow chart. As two children with Integrated Management of Childhood Illness (IMCI) pneumonia also had a positive urine culture and one child with IMCI pneumonia had a positive blood culture, the sum of all diagnoses is 431.

A total of 428 patients between 3 months and 5 years of age were enrolled in the study. Figure 1 shows a flow chart of screening procedures. The most common symptom presented at enrolment was cough, reported in 279/428 (65.2%, 95% CI 60.7–69.7) children, while 60/428 (14.0%, 95% CI 10.7–17.3) children met the IMCI criteria for non‐severe pneumonia, that is cough or difficult breathing with an increased respiratory rate for age. A pathogen detected by blood culture was identified in 6/428 (1.4%, 95% CI 0.3–2.5). A total of 401/428 (93.7%, 95% CI 91.4–96.0) children provided a urine sample. Of these, 24/401 (6.0%, 95% CI 3.7–8.3) had a positive urine culture. Details of the bacteria identified in cultures are presented in Table S1. Table 1 shows additional background details on all enrolled by illness classification.

**Table 1:** Clinical characteristics of 428 children 3–59 months with malaria‐negative fever

	No IMCI criteria of bacterial infection (*N* = 341)	IMCI pneumonia^a^ (*N* = 60)	Urine culture positive (*N* = 24)	Blood culture positive (*N* = 6)
Age <12 months *n* (%, 95% CI) *N* = 174	126 (72.4, 65.8–79.0)	31 (17.8, 12.1–23.5)	16 (9.2, 4.9–13.5)	4 (2.3, 0.1–4.6)
Age 1–5 years *n* (%, 95% CI) *N* = 254	215 (84.6, 80.2–89.1)	29 (11.4, 7.5–15.3)	8 (3.1, 1.0–5.0)	2 (0.8, −0.3 to 1.9)
Females *n* (%, 95% CI)	184 (54.0, 48.7–59.2)	24 (40.0, 27.6–52.4)	12 (50.0, 30.0–70.0)	5 (83.3, 53.5–113.1)
Temp > 37.5 *n* (%, 95% CI)	142 (41.6, 36.4–46.8)	44 (73.3, 62.1–84.5)	8 (33.3, 14.5–52.2)	4 (66.7, 28.9–104.4)
Days with fever mean (median)	3.3 (3)	2.9 (3)	3.1 (3)	5.5 (5.5)
Admitted *n* (%, 95% CI)	8 (2.3, 0.7–3.9)	17 (28.3, 16.9–39.7)	0	1 (16.7, −13.1 to 46.5)

IMCI, Integrated Management of Childhood Illness.

a As two children with IMCI pneumonia also had a positive urine culture and one child with IMCI pneumonia had a positive blood culture, the sum of all diagnoses is 431.

Table 2 provides information on mean and median values of haemoglobin, CRP and WBC depending on illness classification. WBC did not differ significantly between children with IMCI pneumonia/positive urine or blood culture (mean 14.0 × 10^9^, 95% CI 12.0–16.0 × 109, median 13.5 × 10^9^) and those without signs of bacterial infection (mean 12.0 × 10^9^, 95% CI 11.4–12.7 × 109, median 11.5 × 10^9^). CRP was significantly higher in children with IMCI pneumonia/positive urine or blood culture (mean 41.0 mg/l, 95% CI 28.3–53.6 median 16 mg/l) *vs*. children without any signs of bacterial infection (mean 23.8 mg/l, 95% CI 17.8–27.8, median 7 mg/l).

**Table 2:** Haemoglobin, C‐reactive protein and white blood cell median and mean values by illness classification

	No IMCI criteria of bacterial infection (*N* = 341)	IMCI pneumonia (*N* = 60)^a^	Urine culture positive (*N* = 24)	Blood culture positive (*N* = 6)
Haemoglobin g/dl mean (median)	11.1 (11.3)	10.4 (10.6)	10.5 (10.7)	10.6 (10.9)
CRP mg/l mean (median)	23.8 (7)	40.1 (13)	45.0 (24)	29.5 (22)
WBC ×10^9^/l mean (median)	12.0 (11.5)	13.8 (13.5)	14.7 (13.4)	9.2 (8.1)

IMCI, Integrated Management of Childhood Illness.

a As two children with IMCI pneumonia also had a positive urine culture and one child with IMCI pneumonia had a positive blood culture, the sum of all diagnoses is 431.

### C‐reactive protein and bacterial infection

Overall, 256/428 (59.8%, 95% CI 55.2–64.5) children had a CRP value of less than 20 mg/l. Of these, 215/256 (84.0%, 95% CI 79.5–88.5) were children with no signs of bacterial infection, that is no positive blood or urine culture and no IMCI pneumonia. Applying a cut‐off value of CRP at 20 mg/l, the negative likelihood ratio for either a positive blood or urine culture or IMCI pneumonia was 0.71 (95% CI 0.57–0.87). The negative likelihood ratio for a positive urine culture with a CRP below 20 was 0.55 (95% CI 0.33–0.90) and for a positive blood culture 0.64 (95% CI 0.24–1.70). Nevertheless, nine children with a positive urine culture had a CRP below 20 mg/l and two children with a positive blood culture had a CRP below 5 mg/l. None of these presented with any severity signs as defined in the IMCI algorithm, and the fever had reportedly been present for at least two days.

Of all enrolled, 33/428 (7.7%, 95% CI 5.2–10.2) had a CRP level above 80 mg/l. Twelve of these 33 (36.7%, 95% CI 20.3–53.1) had some sign of bacterial infection (positive urine or blood culture or IMCI pneumonia). Repeat CRP tests were carried out in 29 patients, and 12 (41.4%) of these showed a similar result of >200 mg/l in both tests.

**Figure 2:**
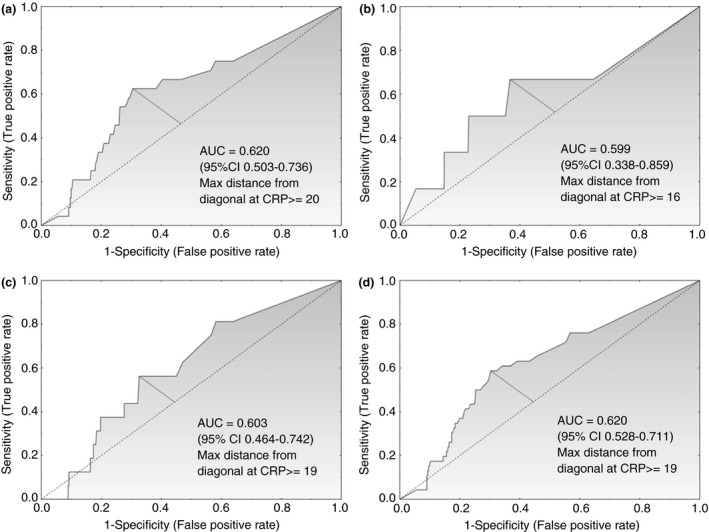
ROC curves for C‐reactive protein (mg/l) in relation to (a) positive urine culture, (b) positive blood culture, (c) IMCI pneumonia and (d) all suspected bacterial infections.

The ROC analysis suggested a cut‐off for CRP of 19 mg/l to differentiate between children with and without signs of bacterial infection, for example positive urine/blood culture or IMCI pneumonia, but the area under the curve (AUC) was low at only 0.62. Similar cut‐offs and AUC values were yielded in separate ROC analysis for blood cultures, urine cultures and IMCI pneumonias, respectively (Figure 2).

### White blood cell density and bacterial infection

**Figure 3:**
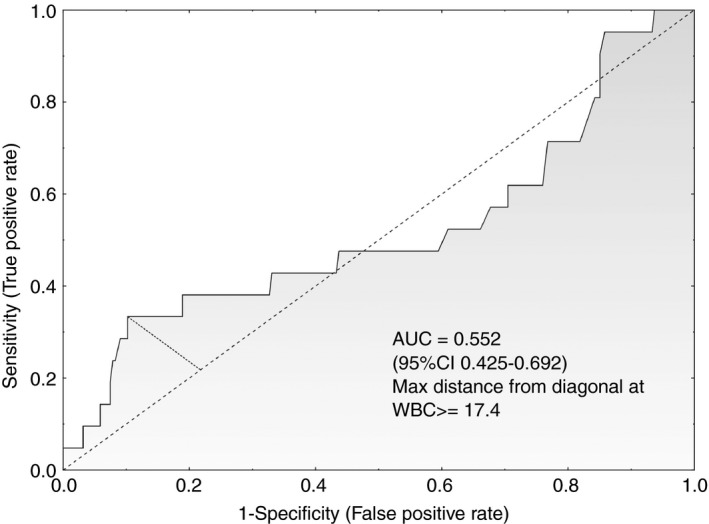
ROC curve for white blood cells (×10^9^) in relation to all suspected bacterial infections.

Seventeen patients had a WBC of above 20 × 10^9^/l. Most of these (13/17, 76.5%, 95% CI 56.3–96.7) had no signs of bacterial infection. There were no tendencies of higher WBC counts in children with positive urine or blood culture nor among children with IMCI pneumonia, and the AUC in ROC analysis was low at 0.52 (Figure 3).

Based on the cut‐off values for CRP and WBC identified in the ROC analysis, sensitivity, specificity and positive and negative predictive values (NPV) were calculated (Table 3). For both CRP and WBC, NPV reached above 80% – mainly explained by the low prevalence of positive cultures – while positive predictive values were all less than 35.9%.

**Table 3:** Sensitivity, specificity, positive predictive value (PPV) and negative predictive value (NPV) of CRP 19 mg/l and WBC <5.0^a^ or >17.4 × 10^9^ to predict paediatric infections

Variable	Cut‐off point	Illness cause	Sensitivity (%, 95% CI)	Specificity (%, 95% CI)	PPV (%, 95% CI)	NPV (%, 95% CI)
CRP	19 mg/l	All	44.6 (33.2–56.6)	78.5 (73.0–83.1)	35.9 (26.3–46.6)	84.0 (78.8–88.1)
		UTI	57.1 (34.4–77.4)	78.4 (72.6–83.1)	17.9 (9.9–29.6)	95.7 (91.7–97.9)
		Bacteraemia	50.0 (9.2–90.8)	78.5 (73.0–83.1)	3.3 (0.6–12.1)	99.1 (96.4–99.8)
		IMCI pneumonia	37.5 (24.3–52.7)	78.5 (73.0–83.1)	23.4 (14.8–34.7)	87.8 (82.8–91.5)
WBC	<5.0 or >17.4 × 10^9^	All	27.8 (14.8–45.4)	83.7 (77.9–88.3)	22.7 (12.0–38.2)	87.1 (81.4–91.2)
		UTI	40.0 (13.7–72.6)	82.9 (77.3–87.4)	9.1 (3.0–22.6)	97.0 (93.3–98.8)
		Bacteraemia	33.3 (1.8–87.5)	83.7 (77.9–88.3)	2.9 (0.1–16.6)	98.9 (95.5–99.8)
		IMCI pneumonia	23.8 (9.1–47.5)	83.7 (77.9–88.3)	12.8 (4.8–28.2)	91.6 (86.5–95.0)

IMCI, Integrated Management of Childhood Illness.

a As some bacterial infections present with a low WBC, we also used a lower cut‐off limit for WBC.

## Discussion

Point‐of‐care bring a number of advantages that are particularly relevant to resource‐poor countries such as their simplicity and independence from distant laboratories or complex quality control systems. However, PoC tests come at a cost, both financial and in health worker time, and their use must be justified by evidence. In this study, we found that the measurement of white blood cells and CRP did not facilitate diagnosis of bacterial illness in children with malaria‐negative fever in Tanzania. As a single indicator of bacterial infection, both WBC and CRP did not provide sufficient accuracy to justify their use in assessing children with non‐severe febrile illness.

Assessment of white blood cells failed to show any significant difference between children with and without suspected bacterial infections. The use of white blood cell differential count has a long history of use as a diagnostic marker of bacterial illness but in recent studies has shown poor specificity and sensitivity in a review of potential biomarkers to identify serious bacterial illness 19. This study confirms the limited diagnostic use of WBC testing in paediatric fevers in resource‐poor settings.

In comparison, CRP values were generally higher among children with suspected or confirmed bacterial illness than among children without any signs of bacterial illness. The cut‐offs in ROC analysis were around 20 mg/l for IMCI pneumonia as well as for confirmed positive urine or blood cultures, but as in other studies, the AUC values were too poor for the cut‐off value to be trusted 14, 20. Notably, the suggested cut‐off and AUC values were similar for IMCI pneumonia and confirmed positive blood cultures. This is despite the fact that the IMCI algorithm for pneumonia used in 2011/12 is known to overdiagnose pneumonia. In other studies, the use of CRP assessment has proven useful to predict radiologically confirmed pneumonia, and even to differentiate viral from bacterial causes 8, 12, 21, 22. Results from these previous studies have suggested higher cut‐off values of CRP in pneumonia than the one in this study, the value perhaps being lower due to the incomplete availability of X‐rays.

The current IMCI algorithm does not include any suggestions on how to diagnose urinary tract infections although these infections contribute to more than 5% of paediatric fevers 9. Urine dipsticks have been suggested as part of a refined algorithm to assess paediatric fevers in low‐resource settings, but sampling of urine is time‐consuming and samples are easily contaminated 23. Considering the relatively high NPV of CRP for urinary tract infections, a combination of urine dipstick and CRP assessment may be useful to improve diagnostic accuracy of paediatric urinary tract infections and in particular contribute to the identification of children with upper urinary tract infections and risks of renal scarring 24.

The suggested cut‐off in ROC analysis for CRP levels to consider bacterial illness was 19 mg/l. This is similar to the proposed cut‐off value of 20 mg/l to rule out severe bacterial infection in a systematic review of paediatric fevers 25. Still, some children who were admitted had a normal CRP level, for example below 5 mg/l, despite being sick enough to require admission. While none of these children had features specific to bacterial disease and thus may have suffered viral infections, it highlights the importance of not relying on a single marker in the assessment of a sick child but rather combines different diagnostic tools to decide on how the illness should be managed. The low prevalence of positive blood cultures in outpatients will inevitably result in very low positive predictive values of anything other than a very accurate test and makes it unlikely that any single symptom or biomarker alone can guide treatment when assessing a febrile child.

### Study limitations

We excluded patients with a positive malaria RDT in this study as earlier studies have shown the inability of CRP/WBC to differentiate between malaria and bacterial infections despite tendencies of higher values in bacterial infections 26. Urine cultures were only performed on children with a dipstick positive for leucocytes and/or nitrate, and we may thus have missed some urinary tract infections without pathologic results on urine dipsticks. Furthermore, as RDTs may remain positive after the malaria illness has resolved, there is a risk that some patients with a positive RDT also suffered bacterial/viral illness and were incorrectly excluded. The use of IMCI pneumonia as a proxy for bacterial pneumonia diagnosis is known to overestimate pneumonia but was used as high‐quality X‐rays were only available for 50% of included children. The rather low percentage of IMCI pneumonias compared to other studies may be partly due to an intense training of research staff to count respiratory rate properly but may also be influenced by the inclusion criteria requiring fever today or yesterday to enter the study, possibly decreasing the overall severity of illness in the group. As serological tests were not carried out, some patients may have suffered from zoonoses in which CRP/WBC levels remain unknown 27.

Furthermore, the normal distribution of CRP may differ between different areas and also seem to be affected by malaria endemicity as well as genetic factors that may limit generalisability and call for local cut‐off recommendations 28, 29.

The Afinion CRP analyser can only work in a temperature range between 15 and 32°C, which obviously constitutes a limitation for PoC analysis of CRP in tropical settings in general.

## Conclusions

We conclude that assessment of WBC count had a limited value in the identification of children with bacterial infections in non‐malarial fevers presenting to an outpatient department. Both CRP and WBC measurements resulted in positive predictive values that were too low to be used as a single tool for treatment decisions.

## Supporting information


**Table S1.** Bacterial aetiologies in blood cultures and urine cultures.
