# Supplementary material for: Point‐of‐care assessment of C‐reactive protein and white blood cell count to identify bacterial aetiologies in malaria‐negative paediatric fevers in Tanzania
Source: Trop Med Int Health. 2016 Dec 28;22(3):286–93. doi: 10.1111/tmi.12823 (PMC5336187; doi:10.1111/tmi.12823)
Supplement: Supplementary file 1 — Table S1. Bacterial aetiologies in blood cultures and urine cultures. [file TMI-22-286-s001.docx]

**Supplementary table 1: Bacterial aetiologies in blood cultures and urine cultures**

| **Sex and age** | **Blood pathogen** | **Days ill** |  | **Sex and age** | **Urine pathogen** | **Days ill** |
| --- | --- | --- | --- | --- | --- | --- |
| Female, 3mo | *Gr A Beta haemolytic streptococci (A)* | 4 |  | Female, 4mo | *E. coli* | 3 |
|  |  |  |  | Female, 5mo | *E. coli* | 2 |
| Male, 8mo | *S. pneumonia* | 7 |  | Female, 5mo | *E. coli* | 3 |
| Female, 9mo | *E. coli* | 5 |  | Female, 6mo | *E. coli* | 3 |
| Female, 10m | *S. pneumonia* | 4 |  | Male, 6mo | *E. coli* | 3 |
| Female, 3yrs | *S. typhi* | 7 |  | Male, 7mo | *E. coli* | 3 |
| Female, 4yrs | *S. typhi* | 7 |  | Female, 7mo | *E .coli* | 3 |
|  |  |  |  | Male, 7mo | *E .coli* | 3 |
|  |  |  |  | Male, 8mo | *E. coli* | 3 |
|  |  |  |  | Male, 8mo | *E. coli* | 2 |
|  |  |  |  | Female, 9mo | *E. coli* | 3 |
|  |  |  |  | Male, 9mo | *E. coli* | 7 |
|  |  |  |  | Male, 10mo | *E. coli* | 2 |
|  |  |  |  | Male 10mo | *E. coli* | 3 |
|  |  |  |  | Female,10m | *E. coli* | 3 |
|  |  |  |  | Male, 11mo | *E. coli* | 5 |
|  |  |  |  | Male, 14mo | *E .coli* | 4 |
|  |  |  |  | Male, 15mo | *E. coli* | 2 |
|  |  |  |  | Female, 17mo | *Klebsiella* | 3 |
|  |  |  |  | Female, 22mo | *S. aureus* | 3 |
|  |  |  |  | Female, 36mo | *Klebsiella* | 3 |
|  |  |  |  | Female, 40mo | *E. coli* | 2 |
|  |  |  |  | Male, 42mo | *Enterococci* | 3 |
|  |  |  |  | Female, 56mo | *Enterococci* | 3 |
|  |  |  |  |  |  |  |
